# The Potential of Silk and Silk-Like Proteins as Natural Mucoadhesive Biopolymers for Controlled Drug Delivery

**DOI:** 10.3389/fchem.2015.00065

**Published:** 2015-11-26

**Authors:** Amanda E. Brooks

**Affiliations:** Department of Pharmaceutical Sciences, North Dakota State UniversityFargo, ND, USA

**Keywords:** silk, sericin, mucoadhesive, drug delivery, biopolymers, aggregate silk

## Abstract

Drug delivery across mucus membranes is a particularly effective route of administration due to the large surface area. However, the unique environment present at the mucosa necessitates altered drug formulations designed to (1) deliver sensitive biologic molecules, (2) promote intimate contact between the mucosa and the drug, and (3) prolong the drug's local residence time. Thus, the pharmaceutical industry has an interest in drug delivery systems formulated around the use of mucoadhesive polymers. Mucoadhesive polymers, both synthetic and biological, have a history of use in local drug delivery. Prominently featured in the literature are chitosan, alginate, and cellulose derivatives. More recently, silk and silk-like derivatives have been explored for their potential as mucoadhesive polymers. Both silkworms and spiders produce sticky silk-like glue substances, sericin and aggregate silk respectively, that may prove an effective, natural matrix for drug delivery to the mucosa. This mini review will explore the potential of silk and silk-like derivatives as a biocompatible mucoadhesive polymer matrix for local controlled drug delivery.

## Introduction

The rising need for tissue compatible adhesives is expected to generate a $38 billion global market by 2017 (Bré et al., 2013). A subset of this market is being driven by the pharmaceutical industry. Localized transmucosal drug delivery constitutes a large and growing share of the market, with an estimated value of $6.7 million (U.S.) in 2006 (Andrews et al., 2009) and $2.91 billion (global) in 2013 (Micromarket Monitor^1^). Based on a compound annual growth rate of 6.8%, global transmucosal drug delivery is projected to be a $4.05 billion market segment by 2018 (Micromarket Monitor). This growing market is demanding a new and diverse set of polymers. Local drug delivery and retention, particularly at a biological surface, can often be accomplished through the use of bioadhesive polymers. Mucoadhesives, a class of bioadhesives, serve a critical niche in transmucosal drug delivery as the unique environment at the mucosal surface requires altered drug formulations. The mucosal membrane is typically composed of a specialized epithelial cell layer covered with mucin to facilitate gas and nutrient exchange (Yu et al., 2014). The physiological function of the mucosal membrane can be exploited to facilitate pharmaceutical dosing. Mucoadhesive polymers, including both synthetic and natural polymers, have generated intense and growing interest in the past decades (Grabovac et al., 2005; Khutoryanskiy, 2011; Mythri et al., 2011). In addition to a host of synthetic polymers, prominently featured in the literature are chitosan, alginate and cellulose derivatives. Recently, several silk and silk-like derivatives have been evaluated for their adhesive properties. This mini review will describe the mucoadhesive properties of silk and silk-like derivatives that justify them being explored as biocompatible mucoadhesive polymer matrices for localized, controlled transmucosal drug delivery.

## Mechanisms of mucoadhesion

A variety of mucous membranes exist throughout the human body to lubricate and protect the interface between the internal and external environments of the body. Several good reviews describing the characterization and central role of mucin in mucous membranes are available and will not be further reviewed here except to say that the mucin component of the mucous membrane forms a glyocoprotein gel-like network that proves critical to mucoadhesion (Marriott and Gregory, 1990; Smart, 2005; Andrews et al., 2009; Khutoryanskiy, 2011; Yu et al., 2014). Although the precise mucin organization and identification may vary based on mucosal location (i.e., nose, eye mouth, stomach, intestine, vagina), there are six main theories of mucoadhesion with many principles of mucoadhesion remaining consistent: (1) the wetting theory, which describes mucoadhesion as a product of the intermolecular interactions and interfacial tension between the mucosal surface and the adhesive; (2) the mechanical interlocking theory, which proposes that mucoadhesion results from the mechanical interlocking of the adhesive and features of the substrate surface; (3) the electronic transfer theory, where electrons transfer between the adhesive and the surface creating critical electrostatic forces; (4) the diffusion interpenetration theory, which describes mucoadhesion as a result of the interpenetration and entanglement of polymer and mucin chains dominated by electrostatic attractions; (5) the adsorption theory, which describes mucoadhesion as being an accumulation of primary (i.e., ionic and covalent) and secondary (i.e., van der Waals forces, hydrogen bonding, electrostatic attraction, and hydrogen bonds) bond formation; and (6) the fracture theory, which does not offer a chemical or molecular explanation of mucoadhesion but simply relates the adhesive strength to that necessary to separate the adhesive and mucous membrane (Smart, 2005; Andrews et al., 2009; Shaikh et al., 2011; Tangri, 2011; Yu et al., 2014). Importantly, the underlying mechanism of mucoadhesion is not completely clear and may result from a combination of these theories (Smart, 2005); furthermore, the adhesive strength and consequently the utility of different mucoadesive polymers is not the same for all mucous membranes (Accili et al., 2004). Notably, certain mucoadhesive polymers, including natural biopolymers, may have altered degradation in the GI tract due to the presence of the microbiome or other pathological conditions [i.e., inflammation, ulcerative colitis, etc. (Seves et al., 1998; Hua et al., 2015)]. Thus, awareness of the theories is essential to the design of mucoadhesive polymers for drug delivery systems.

## Mucoadhesive polymers

Regardless of the specific mucoadhesive mechanism, there are some promising candidate polymers in development for drug delivery. General characteristics and classifications of mucoadhesive polymers are presented in this review with examples to illustrate their utility as drug delivery systems opposed to an in depth discussion of all possible polymers.

### Characteristics of polymers

The appropriate polymer choice for local mucoadhesive drug delivery relies on a combination of (1) the polymer's chemical reactivity and stereochemistry, (2) its molecular weight and concentration, (3) its side group flexibility and steric hindrance, and (4) its ability to swell and adhere to tissues under moist or high humidity conditions (Andrews et al., 2009; Shaikh et al., 2011; Tangri, 2011). Commonly used mucoadhesive polymers generally have polar groups (hydroxyl, carboxyl, amide, sulfate) available for interaction with mucin as well as molecular weights that fall in the range of 10^4^ Da to 4 × 10^6^ Da to facilitate the interaction (Smart, 2005; Andrews et al., 2009). Polymers that fall on the upper end of this range may not have sufficient flexibility to swell and adhere while those that are below this range will only form weak adhesives and readily dissipate. First generation or non-specific mucoadhesive polymers, whether synthetic or natural, are generally hydrophilic with functional groups that allow for hydrogen bonding and electrostatic interactions. Alternatively, second generation mucoadhesive polymers such as lectins (Clark et al., 2000; Lehr, 2000; Haas and Lehr, 2002; Kim et al., 2015), invasins, thiolated polymers (Bernkop-Schnürch et al., 2004b; Cevher et al., 2008a,b), antibodies, and other proteins (Woodley, 2001; Bravo-Osuna et al., 2007) are developed to facilitate specific interactions and overcome biological barriers (Carvalho et al., 2010). A review of synthetic mucoadhesive polymers will not be presented in this mini-review, but instead the reader is referred to several reviews on the subject (Grabovac et al., 2005; Ludwig, 2005; Salamat-Miller et al., 2005; Valenta, 2005; Catron et al., 2006; Andrews et al., 2009; Carvalho et al., 2010; Mythri et al., 2011; Yu et al., 2014).

#### Natural polymers

In this synthetic landscape, a host of natural polymers have been explored as mucoadhesive drug carriers (Ceulemans et al., 2002; George and Abraham, 2006; Wittaya-areekul et al., 2006; Kalu et al., 2007) and are often preferred for biomedical applications due to their reputation for “green” processing, renewability, and biocompatibility (Ngwuluka et al., 2014). Notably, biocompatibility, specifically immunocompatibility, may be a product of purity, which could be challenging for natural sources (Lehr, 2000). Many naturally mucoadhesive polymers are very large polymeric proteins and have repetitive patterned structural elements organized in a structural hierarchy, particularly in the silks (Table 1). The mechanism of mucoadhesion for many of these natural polymers seems to begin with physical entanglement and ultimately relies on the use of secondary, non-covalent bonds, similar to other first generation mucoadhesive polymers. However, common chemical modifications (e.g., DOPA, etc.) are also found in natural mucoadhesive polymers and offer important insight in to mucoadhesion (Lee et al., 2002; Bré et al., 2013). These characteristics provide a foundation that allows for tunable drug release and permeability based on secondary structural elements, a distinct advantage over many synthetic alternatives.

**Table 1:** **Comparison of the repetitive primary structural elements of natural adhesive polymers**.

**Natural polymer**	**Amino acid motif/Chemical structure**	**Ecological purpose**		**Adhesion strength**	**References**
Sericin	(SSTGSSSNTDSNSNSVGSSTSGGSSTYGYSSNSRDGSV)_n_	Sticky outside coating	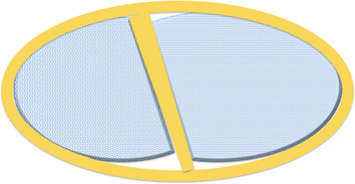	4.1 ± 2 N	Ahn et al., 2001
Silkworm firboin	GAGAGS, GX_n_, where X = A, Y, V	Core of silk fiber	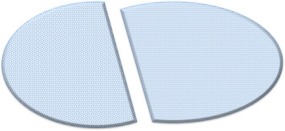	54 mN or 146.6 mN/cm^2^	Jiang et al., 2006; Kundu et al., 2008a
Aggregate silk	Gly-rich (64-mer), XPGXG (36-mer,) GGX/NXNXN (33-mer)	Aqueous glue for web	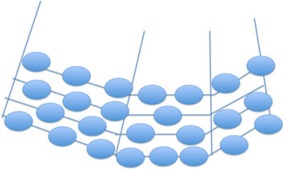	0.1–0.4 mN	Sahni et al., 2011; Vasanthavada et al., 2012; Opell et al., 2013
Piriform silk	QQSSVA, PXPXP	Attachment cement	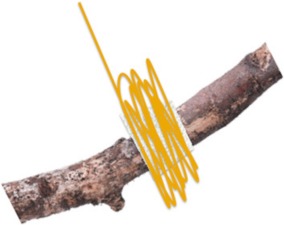	39.8 ± 8.9 mN	Perry et al., 2010; Grawe et al., 2014; Wolff et al., 2015
Caddisfly silk	*O*-phospho-ser cluster (SX)_n_ where X = V, L, I, R; and *n* is 2–6	Underwater cement for protective case	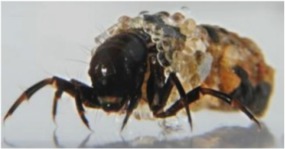	32.7 ± 6.6 MPa (stress at fracture)	Ohkawa et al., 2013; Lane et al., 2015
Chitosan	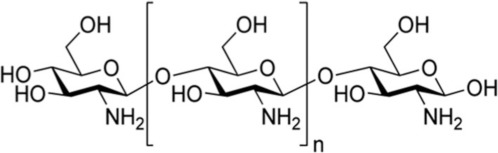	Shellfish	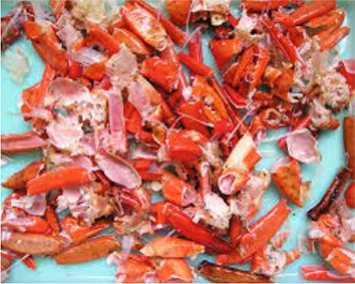	32.4 ± 14.5 mN 3.9–6.7 mN/cm^2^	Bernkop-Schnürch and Freudl, 1999; Lehr et al., 1992

*Note that the values for adhesion strength cannot be compared due to different techniques used to gather the data*.

##### Chitosan

Chitosan is perhaps the most studied natural mucoadhesive polymer and has been extensively considered for drug delivery due to its mucoadhesive and stimuli responsive nature. Interestingly, without modification, chitosan, a derivative of shellfish, has an adhesive force that exceeds both carboxymethylcellulose and polycarbophil, two of the most common synthetic mucoadhesives used in drug delivery (Bravo-Osuna et al., 2007; Ngwuluka et al., 2014). This versatile natural polymer exemplifies the mucoadhesion of the amino functionality of a cationic polymer to the sialic groups of mucin through electrostatic interactions at physiological pH (Carvalho et al., 2010; Kim et al., 2015) and has been used to deliver many drugs [e.g., metronidazole vaginally (Valenta, 2005), AZT nasally (Barbi et al., 2015), and pilocarpine ocularly (Li and Xu, 2002), etc.]. Unfortunately, despite its promise, there are no FDA approved chitosan drug delivery systems currently (Kean and Thanou, 2010; Rodrigues et al., 2012; Thakur and Thakur, 2015). Like PEG, chitosan can be readily functionalized to improve its mucoadhesion (Andrews et al., 2009). During the delivery of rhodamine or calcitonin, thiolation of chitosan has been shown to (1) increase its mucoadhesive strength by allowing disulfide bridges with mucous glycoprotein cysteine residues, (2) promote mucous permeation, and (3) prevent protease activity by sequestering zinc and magnesium, important cofactors for protease activity (Bernkop-Schnürch et al., 2004a,b; Grabovac et al., 2005; Bravo-Osuna et al., 2007; Cevher et al., 2008a). Importantly, thiolation may not be appropriate for all mucoadhesive drug delivery systems due to formation of stable, yet short lived, disulfide bonds with mucin, increasing mucoadhesion up to 130-fold when FITC-dextran was delivered (Bernkop-Schnürch et al., 2004b; Shaikh et al., 2011; Kim et al., 2015). Chitosan has also been complexed with catechol, a side chain of DOPA, to increase *in vivo* retention and release of orally-delivered insulin for up to 10 h, as opposed to less than 3 h for chitosan alone (Kim et al., 2015). Alternatively, when chitosan was mixed with a catechol-containing compound, hydrocaffeic acid, and tested in a rabbit intestine, swelling could be decreased with a corresponding increase in mucoadhesion and release of hydrocaffeic acid (Xu et al., 2012). The ability to modify chitosan and create the specificity of binding, characteristic of a second generation mucoadhesive, will provide significant advances in the ability to use chitosan for transmucosal drug delivery, particularly for membranes with high turn-over.

##### Viscoelastic spider silk glues

Unlike chitosan, viscoelastic spider silk glues, while long recognized for their adhesive properties, have yet to find a niche in mucoadhesive drug delivery. Although previous research efforts to use spider silk as a drug carrier (Hofmann et al., 2006; Lammel et al., 2010; Gomes et al., 2012) have focused on the mechanically robust solid major ampullate and flagelliform fibers, aggregate silk glue, and even the piriform cement, are promising mucoadhesive polymer alternatives (Opell and Hendricks, 2009; Sahni et al., 2010). Recently, the primary sequences of both piriform (Perry et al., 2010) and aggregate silk proteins have been determined and the presence of chemical binders such as DOPA to provide adhesive strength is notably lacking (Sahni et al., 2011; Jones et al., 2015). Nevertheless, according to the Dahlquist criteria for adhesives, materials with robust adhesion should have a Young's modulus lower than 100 kPa; atomic force microscopy has measured the average Young's modulus of aggregate silk glue to be 70 ± 47 kPa (Torres et al., 2014). Thus, the fundamental basis of this robust adhesion may lie in the structural hierarchy, a proteinaceous block co-polymer composed of two proteins, ASG1 and ASG2, with a repetitive amino acid motif architecture (Choresh et al., 2009; Vasanthavada et al., 2012; Wolff et al., 2015). Additionally, similar to other natural and synthetic mucoadhesive polymers, ASG1 has a high percentage of charged amino acids, while ASG2 has a motif structure similar to elastin providing the mobility necessary for swelling and interaction with mucin (Choresh et al., 2009; Sahni et al., 2014). This balance of adhesion and elasticity arising from a composite material is a common theme in natural bioadhesives (Lee, 2010) and likely leads to effective dissipation of mechanical forces and mucoadhesion. In fact, the structural hierarchy and heteromeric composition of these silk glues produces an anisotropic material that may limit crack propagation, effectively increasing adhesion strength (Wolff et al., 2015). In addition to the protein composition and organization, aggregate silk has a viscous glycoprotein core surrounded by an aqueous solution of salts (Sahni et al., 2014). Importantly, studies have shown that the glycoprotein component of aggregate silk glue shares several characteristics with mammalian mucin molecules (Choresh et al., 2009). Currently, aggregate silk has not been specifically assessed for mucoadhesion; however, its ability to adhere in a high humidity environment makes it a potentially useful polymer. In fact, as humidity increases, the adhesive strength of aggregate silk glue also increases (Opell et al., 2013; Amarpuri et al., 2015). Although not specifically studied for aggregate silk, a pH gradient may also solidify aggregate silk providing more strength and stability similar to major ampullate silk (Breslauer et al., 2009; Andersson et al., 2013). Further contributing to its potential as a mucoadhesive polymer, the charge of ASG1 has a strong similarity to chitin-binding proteins and should react similarly to changes in pH (Sahni et al., 2014). The composition and structural organization of aggregate silk in addition to its environmentally dependent behavior could prove a critical clue in the use of a silk-based mimetic glue as a mucoadhesive polymer, specifically for drug delivery.

In addition to a viscous aggregate silk glue, spiders also produce piriform silk, a cemented attachment disk, as a solid fiber and fibrous cement composite (Wolff et al., 2015). The cement component, which has a high content of polar and charged amino acids similar to other mucoadhesives (Blasingame et al., 2009; Geurts et al., 2010; Grawe et al., 2014), acts as a viscoelastic fluid capable of filling surface microarchitecture to provide a high contact area that heavily relies on hydrogen bonding for it adhesive strength (Wolff et al., 2015). The anisotropic organization may again provide a level of robust adhesion not achieved with other non-silk bioadhesives.

##### Silkworm-derived adhesives

In contrast to spiders, silkworms can be farmed, providing a level of accessibility for research and commercialization not currently possible with spider silk adhesives, which rely on recombinant development and is still in its infancy. Silkworms produce a single type fiber with a two chain composite fibroin core and a sericin coating (Zhang, 2002; Yucel et al., 2010). The core fibroin is capable of binding to glycoproteins and proteoglycans (Jiang et al., 2006; Dong et al., 2015). Recently, silkworm fibroin was solubilized and processed as a pH-sensitive hydrogel via electrogelation (e-gel) (no drug was released), demonstrating adhesion likely due to secondary bond interactions (e.g., hydrogen bonds and van der Waals interactions). The authors noted that the promising adhesive strength of these e-gels will be assessed for their mucoadhesive abilities in future studies (Yucel et al., 2010). Other efforts to create new “green” silk-based mucoadhesives have complexed silk fibroin with other synthetic polymers. Recently, solubilized silkworm fibroin was combined with a chemically active polyethylene glycol to provide strong adhesive properties (Serban et al., 2011). At a 20% w/v of silk, the adhesive strength of the composite was greater than that of the commercially available CoSeal tissue sealant. Alternatively, silk fibroin was combined with hydroxy propyl methyl cellulose (HPMC) and PEG to create a robust mucoadhesive film for transmucosal drug delivery, although the study did not actually provide any specific drug release kinetics (Kundu et al., 2008a).

The sericin fraction of silkworm silk, which constitutes 25–30% of the silk protein and is routinely discarded during silkworm cocoon processing, can also be blended with a variety of different polymers including sodium alginate (De, 2003; Khandai et al., 2010), polyvinylalchol, polyacrylic acid, and acrylamide to delay and control the release of a pharmaceutical (Ahn et al., 2001; Zhang, 2002; Khandai et al., 2010). Importantly, sericin separated from the fibroin core is inherently adhesive as well (Teramoto and Miyazawa, 2005; Khandai et al., 2010); however, conjugation of sericin with other polymers is reported to stabilize the structure and mitigate residual immunogenicity (Kundu et al., 2008b).

##### Caddisfly silk

Analogous to the aggregate silk secreted by orb-weaving spiders, caddisflies also secrete an adhesive silk-like protein with impressive strength (Stewart and Wang, 2010; Lane et al., 2015). However, unlike aggregate silk, sericin, chitosan, and the DOPA residue similar to that found in the underwater adhesive of muscles, evidence suggests that the adhesive force of caddisfly silk results from the post translational phosphorylation of serine, L-*O*-phospho-serine (Ser(PO_3_H_2_)) (Ashton et al., 2013). It has been suggested that the caddisfly uses Ser(PO_3_H_2_) providing for very strong adhesion likely due to a combination of covalent-crosslinking and electrostatic interactions (Stewart et al., 2011; Ohkawa et al., 2013); however, the precise mechanism is not clear (Wang et al., 2009). The viscous silk-like substance and adhesive acts as an underwater cement to adhere small stones and pebbles to one another to create a protective case; however, the requirements for adhesion between two hard surfaces may prove to be very different from that required for mucoadhesion. Nevertheless, the underwater performance of the material may prove a compelling reason to consider its mucoadhesive properties.

## Mucoadhesion in drug delivery

Since its inception in the 1980s, mucoadhesion has become an increasingly popular alternative drug delivery platform due to its multiple advantages and the advent of multifunctional polymers. The mechanism of release from the different mucoadhesive polymers is often dependent on the site, the pH, and the polymer's swelling characteristics, but overall release is dominated by diffusion and swelling with the primary advantage of the system derived from increased residence time (Yadav et al., 2010; Fini et al., 2011; Mythri et al., 2011).

### Advantages

#### Residence time

Arguably, the primary advantage of mucoadhesive mediated drug delivery is the increased local residence time at the desired site of action due to improved contact (Woodley, 2001; Carvalho et al., 2010; Mythri et al., 2011; Yu et al., 2014). One of the clearest demonstrations of the effect is in ocular (Dong et al., 2015) applications where silk fibroin has been used to coat liposomes for ocular ibuprofen drug delivery to increase the residence time in the precorneal area of the eye. Similar results can also be obtained by replacing silk fibroin with chitosan (Kim et al., 2015), hyaluronan, or cellulose derivatives (Dong et al., 2015). This feature has recently been demonstrated in preclinical studies of ophthalmic drug inserts to treat external ophthalmic diseases in a canine model, which reduced dosing applications to a single treatment (Baeyens et al., 2002). Examples of enhanced drug delivery via mucoadhesion, although not necessarily with silk, can also be found in oral (e.g., FDA-approved Striant testosterone bucal system), nasal (e.g., insulin), gastrointestinal (e.g., many antibiotics; Batchelor, 2005), and vaginal applications (e.g., progesterone; Donnelly and Woolfson, 2015). Additionally, mucoadhesive gels and gel-like particles with their associated rheological properties decrease the mucous clearance and increase the contact time, effectively reducing dosing frequency and increasing patient compliance (Tangri, 2011; Yu et al., 2014).

#### Enhanced safety and efficacy

Mucoadhesive drug delivery often provides enhanced safety and efficacy rooted in the (1) ability to target the mucosa (Woodley, 2001), (2) improved bioavailability of the drug (Woodley, 2001; Mythri et al., 2011; Shaikh et al., 2011; Tangri, 2011), (3) abundant blood flow associated with mucosal surfaces, which will quicken the onset of action (Tangri, 2011), (4) protection of peptide drugs from protease degradation (Bernkop-Schnürch et al., 2004b), and (5) circumvention of first-pass hepatic metabolism (Andrews et al., 2009). Thiolation has also been reported to enhance penetration of the drug (Bernkop-Schnürch et al., 2004b). The nasal administration of insulin as a bioadhesive powder provides an excellent example of the power of mucoadhesive drug delivery (Nagai et al., 1984).

### Barriers

Unfortunately, several barriers hamper the rapid clinical translation of mucoadhesive drug delivery. Barriers to the implementation of successful mucoadhesive drug delivery systems can be divided into either technical limitations of characterization or more inherent biological obstacles.

#### Mechanical assessment

Evaluation of new mucoadhesive polymers requires both *in vitro* and *in vivo* testing to determine adhesive strength, and yet, as with most *in vivo/in vitro* correlations, there is a disconnect in the methodologies (Khutoryanskiy, 2011). Although there are three main testing methods recognized—tensile tests, shear strength, and peel strength with rheology often being included depending on the proposed application, uniform methodologies have not been established (Andrews et al., 2009; Davidovich-Pinhas and Bianco-Peled, 2010; Khutoryanskiy, 2011; Shaikh et al., 2011; Tangri, 2011; Woertz et al., 2013; Yu et al., 2014). Khutoryanskiy provides a nice review of the various testing methods with a discussion of their advantages and disadvantages (Khutoryanskiy, 2011). Lack of uniform testing tools not only hampers comparison of mucoadhesive polymers and drug delivery systems but also proves a critical logistical barrier to regulatory approval. Additionally, the advent of nanoscale pharmaceutical therapies has left a significant void in the methodologies to assess nanoscale mucoadhesion as opposed to macroscale bulk adhesion (Das Neves et al., 2011).

#### Biological factors

In addition to poor methodologies for assessment, many *in vitro* assessments are inaccurate *in vivo* due to biological factors. Several biological factors can affect the feasibility and effectiveness of mucoadhesive drug delivery. The precise pH and microenvironment at the polymer/membrane interface can significantly impact not only the strength of mucoadhesion but also the choice of mucoadhesive polymer (Smart, 2005; Yadav et al., 2010). Various disease states (e.g., common cold, gastric ulcer, etc.) can also alter the chemical and physical environment of the mucous membrane (Mythri et al., 2011; Tangri, 2011). Prolonged contact with the mucous membrane has also been reported to cause irritation (Tangri, 2011). Ultimately, mucous membrane turnover will eventually impact all mucoadhesive drug formulations (Yadav et al., 2010; Tangri, 2011); however, use of second generation mucoadhesive polymers that target, contact, and/or penetrate underlying cells may enhance the longevity of the adhesive and prove more effective platforms for mucoadhesive drug delivery (Lehr, 2000).

## Conclusion

Mucoadhesion is a promising strategy for targeted, controlled drug delivery. Regardless of the specific molecular mechanism, it may prove more effective than other controlled delivery strategies based on (1) the intimate contact provided by the adhesive with an absorpative membrane, (2) the enhanced retention at the site of action, (3) the potential protection of sensitive biological molecules, and (4) the improved bioavailability. Considering the potential of this drug delivery strategy, development of additional natural mucoadhesive polymers is paramount. Chief among these are spider aggregate and piriform silk, silkworm fibroin and sericin, and caddisfly silk. Based on their recognized biocompatibility (Ngwuluka et al., 2014), utilizing bioinspired silk polymers (i.e., aggregate silk, piriform silk, silkworm fibroin, sericin, and caddisfly silk) may mitigate the immune response while proving effective for controlled drug delivery.

## Author contributions

AB is accountable for all work presented in this manuscript, including concept, research, drafting, revision, and final approval.

## Funding

This work was funded by start up funds from North Dakota State University.

### Conflict of interest statement

The author declares that the research was conducted in the absence of any commercial or financial relationships that could be construed as a potential conflict of interest.

## Footnotes

^1^Micromarket Monitor Global Transmucosal Drug Delivery Market Research Report. Available online at: http://www.micromarketmonitor.com/market-report/transmucosal-drug-delivery-reports-5655777892.html [Accessed August 24, 2015].
